# Notes from Past Meetings of the Lippe Society

**Published:** 1890-07

**Authors:** 


### NOTES FROM PAST MEETINGS OF THE LIPPE SOCIETY.

SMALL-POX.—At a meeting of the society, held January 11th, 1881, Dr. Clark spoke of a case of confluent small-pox then under treatment. Dr. Carleton Smith said he had had a chance to try Carnem. (This preparation was given to Dr. Clark by the late Dr. Bayard, of New York, who received it from the late Dr. Reisig.) The patient's face was covered with pustules. One dose of Carnem was given. The next day the pustules had collapsed, and the patient recovered without the least pitting.

[Dr. Fellger was better qualified to speak upon the effects of vaccination and small-pox than any one whom we have ever met. He had given the subject much thought and many years of study. Hence, his remarks are entitled to deep consideration.]

Dr. Fellger.—A short time ago I had a case of probable small-pox. Malandrinum was given. The next day the pustules were dry.

It is reported in an anti-vaccination journal that a cow had been successfully inoculated with "grease" from the horse, and the cow had every appearance of an attack of small-pox. I have given Variolinum to hundreds of people, and none of them have ever been attacked with small-pox. I obtained the Variolinum

I used from a child with small-pox—the case being perfectly typical of the disease. I also use it for ordinary small-pox. For the confluent variety I use Malandrinum.

In England, where the cows were milked by women, Jenner could get no vaccine. It is found only where men milk the cows, and at the same time groom the horses. They thus carry the horse disease to the cows. In one family, where the father had confluent small-pox, I gave Variolinum as a prophylactic to the others, and not one of them took the disease. I knew of a case of a broker, in this city, who is now well marked from small-pox. When a child he was vaccinated, and became idiotic in consequence. When sixteen years old he had a violent attack of small-pox, after which he recovered his reason.

Variolinum is indicated in small-pox where there is not much pain. The patient can even eat a good meal. The skin looks natural between the pustules. This is a mild form of the disease. It needs scarcely any treatment. Small-pox is dangerous where there is an unnatural color of the skin between the pustules, and the disease takes the confluent form. The appearance of the eyeball is important in this disease in making a prognosis. Where the eyeball is white and natural, there is no danger. But if it be red and much congested it is dangerous. I have just had a case of small-pox in a child, who vomited immense quantities of blood before the pustules appeared. This was controlled by Hamamelis.

VACCINATION.—Dr. Fellger said that vaccination was against common sense. Dr. Jenner, the originator of vaccine, confessed that the cow acquired the cow-pox from the fetlocks of the horse.

Dr. Lippe said that Thuja was a great remedy for diseases of the fetlock in the horse. Thuja also was effective in syphilis. This leaves a field for speculation as to the essential nature of the disease of the fetlock in the horse, from which the cow gets the cow-pox.

Dr. Fellger said he had used Malandrinum with great success in diseased conditions from vaccination.

NOSODES.—Dr. Fellger returned to the subject of nosodes. He said it was absurd to claim that any disease could be cured by its nosode, for, after potentizing the nosode, we cannot be satisfied that it is in the same condition as when first taken from the diseased individual. Thus the syphilitic poison is composed of molecules: the molecules of atoms. When the poison is potentized, the essential character of the molecules is undoubtedly lost, and hence it is not the same substance any longer. Therefore, there can be no certainty that the potentized preparation is the same poison. But if, for argument, we allow that the molecules of the poison can be potentized without change of character, we still are not relieved of the dilemma, for primary syphilis in any one person will make a different set of symptoms from syphilis in another person. The variations are endless. Hence we must then have a potentization of each one of these different kinds of syphilis, which would be impossible. Mercury is a stable substance, always unchanged; it might then be expected to produce identical sets of symptoms on any number of the most different people. Yet its action is different upon every person to whom it is given. How much more, then, must be the individual variations in the case of the poison of syphilis, and if Mercury requires so many different remedies to antidote it, how much more, then, must syphilis need a variety of remedies to treat it. It is folly, then, to expect to treat symptoms with its nosode, and the folly the more apparent when we realize that the character of this nosode is essentially changed in the process of potentizing. The only way, therefore, to use a nosode is to *prove* it on the healthy, like any other drug, and note its symptoms in the regular way. In this manner only can the scope of its action be determined. Dr. Lux was the real founder of isopathy. Dr. Reisig was traveling in Europe. He heard Lac-caninum suggested as a remedy for throat diseases. Reisig investigated it, and found out from its rather meagre pathogenesis that it had a striking relation to the throat. He brought his information to America, and communicated it first to Dr. Bayard. Thus was Lac-caninum introduced to the materia medica. It was afterward elaborately proved by Dr. Samuel Swan, to whom we are indebted for its present development. G. H. C.

